# Review of Glycerol Conversion to Glycerol Carbonate via Alkyl Carbonates: Reflections, Future Perspective and Catalytic Roles of Mixed/Promoted Metal Oxides and Mechanistic Insights from DFT

**DOI:** 10.3390/molecules31152623

**Published:** 2026-07-28

**Authors:** Sakhile T. Dube, Lindelani Q. Qwabe, Holger B. Friedrich

**Affiliations:** 1 School of Chemistry and Physics, University of KwaZulu-Natal, Durban 4000, South Africa; dube.sakhile@mut.ac.za (S.T.D.); friedric@ukzn.ac.za (H.B.F.); 2 Department of Chemistry, Faculty of Applied and Health Science, Mangosuthu University of Technology, 511 Mangosuthu Highway, Durban 4000, South Africa

**Keywords:** glycerol, glycerol carbonate, mixed metal oxides, promoted catalysts, mechanistic insights (DFT), green chemistry

## Abstract

This review summarizes recent progress in the catalytic conversion of glycerol to glycerol carbonate (GC) via transesterification with alkyl carbonates, a sustainable route for valorizing surplus glycerol from biodiesel production. Emphasis is placed on mixed and promoter-modified metal oxide catalysts, which exhibit high activity, selectivity, and stability due to their tunable balance of basic and Lewis acidic sites. Systems such as Mg-Fe, Mg-Al, Mg-Zr, CaO-CeO_2_, and Mg-Ba oxides have achieved over 90% glycerol conversion and 90–96% GC selectivity under mild, often solvent-free conditions. Mechanistic insights, increasingly supported by density functional theory (DFT), reveal that the reaction proceeds via base-assisted glycerol deprotonation, carbonate activation at Lewis acidic centers, and subsequent cyclization to GC. These findings underscore the importance of acid–base cooperation and promoter effects in enhancing turnover frequency, reducing energy barriers, and mitigating catalyst deactivation due to carbonate deposition or leaching. By integrating experimental results with theoretical modelling, this review provides a comprehensive understanding of catalyst design principles, offering guidance for the development of efficient and robust systems for scalable glycerol upgrading.

## 1. Introduction

The expansion of the biodiesel industry, driven by concerns over petroleum depletion in the past decade, has resulted in a substantial surplus of glycerol, which is produced as a by-product at approximately 10% (*w*/*w*) of total biodiesel output [1]. This persistent oversupply on a global scale has created both economic challenges and environmental concerns [2,3]. Thus, led by increased demand for energy, climate change, and energy security concerns, researchers and industries have developed an interest in improvement or in finding an alternative such as purification or converting it into a usable chemical, e.g., glycerol carbonate, propanol, glycidol, formic acid, polyesters, etc. [4,5,6]. Valorization of the glycerol would significantly improve the economic aspect of overall biodiesel production, since the direct utilization of glycerol cannot accommodate such large volumes obtained from biodiesel production [7]. This is because glycerol itself is very cheap, but most applications require additional purification processes [7,8]. Hence, the conversion of glycerol to other valuable chemicals, such as propanediols, glycerol carbonate, acrolein, esters of glycerol, and glyceric acid, would not only generate added value for glycerol but also promote biodiesel production [7,9].

**Scheme 1:**
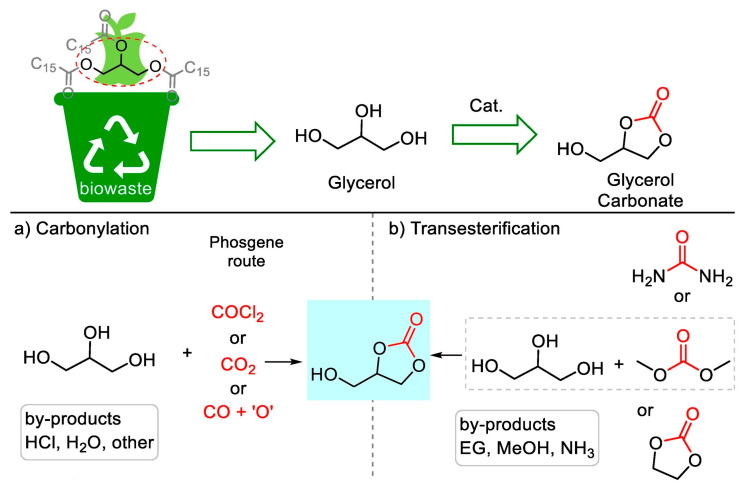
Reaction pathway for the transesterification of glycerol with different reactants leading to the formation of glycerol carbonate [13].

**Figure 1:**
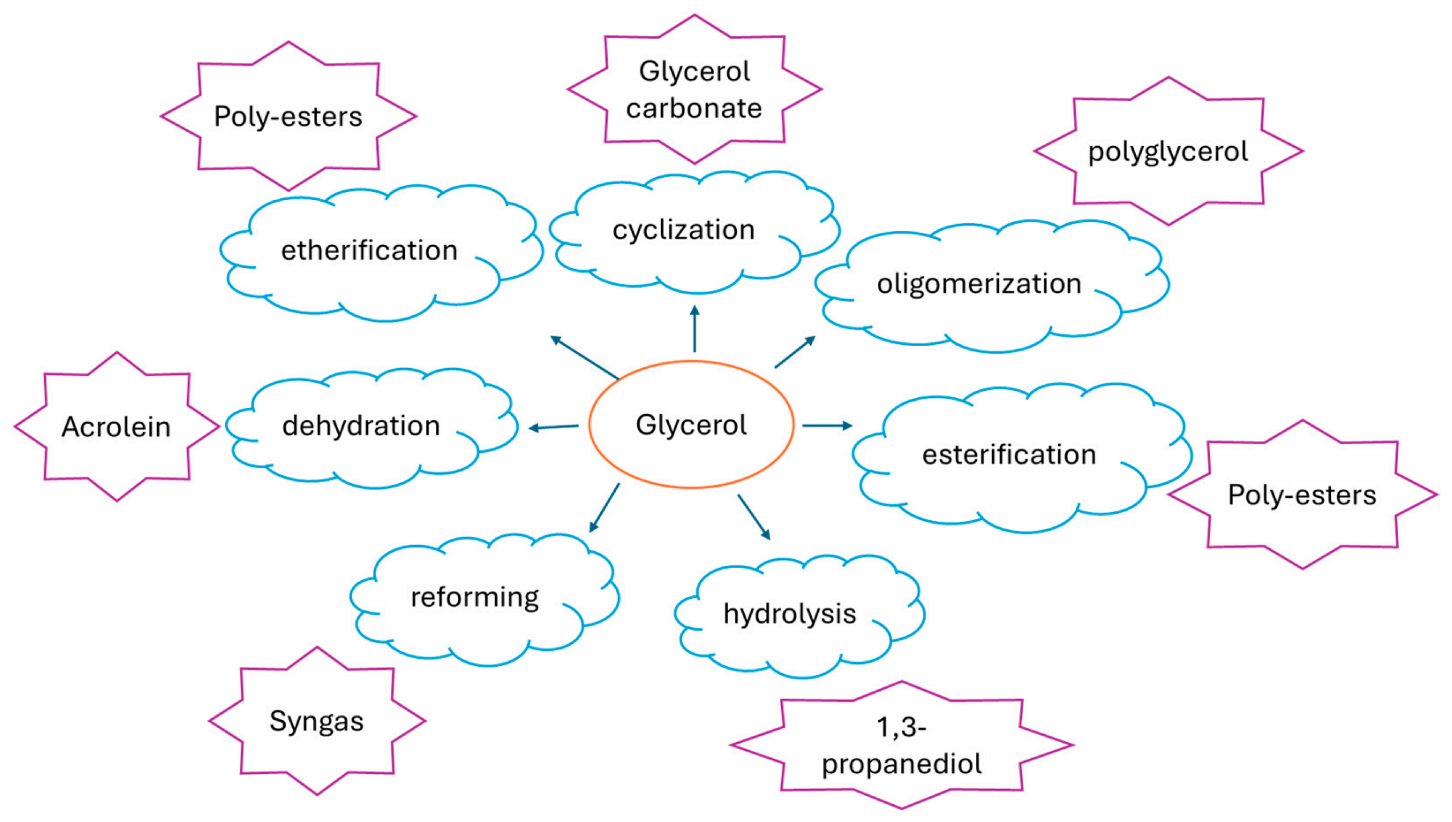
Overview of conversion processes leading to diverse products derived from glycerol.

There are other processes involved in transforming glycerol into multiple derivative compounds, as shown in Figure 1. Converting glycerol into value-added derivatives such as glycerol carbonate (GC) has become a promising route due to its applications as a solvent, electrolyte, polymer precursor, and intermediate in fine chemicals [7,9,10]. Moreover, it has excellent properties, such as low toxicity, biodegradability, low flammability and a high boiling point, which makes it a very attractive chemical for a variety of applications, e.g., as a high-boiling polar solvent [5,10]. Glycerol carbonate from glycerol can be fabricated by different approaches [6]. Useful methods are classified as (i) carbonation of glycerol with phosgene using metallic catalysts, (ii) carbonylation–carbonation reaction between glycerol and urea in the presence of Lewis acid catalysts, (iii) reaction between glycerol and CO_2_ under supercritical conditions, and (iv) lastly, the transesterification of glycerol with dialkyl carbonate over base catalysts [7,11] as shown in Scheme 1. Although glycerol carbonate (GC) holds considerable promise with wide-ranging applications in agrochemicals and polymer chemistry, its market price remains relatively high, at around $8141 per ton (from 2018 to 2024), which restricts large-scale utilization [12]. Consequently, developing cost-effective production routes and identifying high-value applications are essential to fully exploit GC’s potential as a key building block in the emerging bioeconomy [12,13].

Among the possible synthetic pathways, the transesterification of glycerol with dimethyl carbonate (DMC) is considered the most attractive: it is atom-efficient, environmentally benign, and operates under mild conditions. Catalyst development remains central to process efficiency and selectivity. Since the beginning of the 21st century, significant attention has focused on mixed metal oxides (MMOs) [14], promoted oxides [15], and mechanistic understanding through density functional theory (DFT) [16].

**Figure 2:**
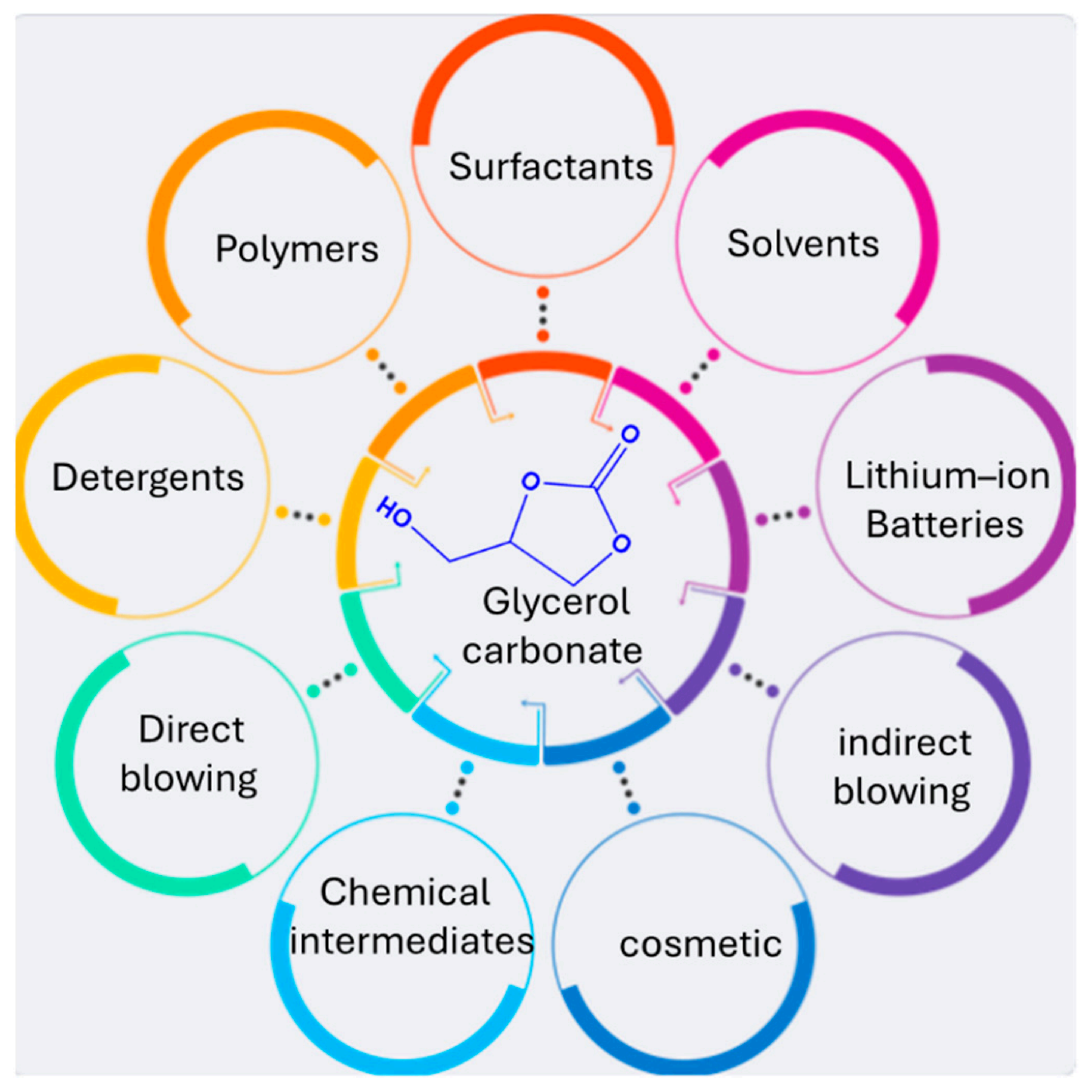
Illustrates the glycerol carbonate uses and applications.

Glycerol carbonate (GC) has emerged as a promising green solvent, precursor to biodegradable polymers, and functional additive in various industrial applications [17]. GC is a compound of high industrial relevance, with applications as a polar aprotic solvent, electrolyte in lithium-ion batteries, intermediate in polymer chemistry, and precursor for glycidol and other fine chemicals, as indicated in Figure 2 [17]. Traditional synthetic pathways to GC, such as phosgene-based routes, glycerolysis with urea, and direct carbonylation with CO_2_, face significant drawbacks, including toxicity, high energy demand, or low selectivity [18,19]. Consequently, the transesterification of glycerol with alkyl carbonates, such as dimethyl and diethyl carbonate, has gained attention for offering enhanced environmental compatibility, milder reaction conditions, and simpler downstream separation [20]. However, the choice of catalyst is a critical determinant, and so far, mixed or promoter-modified metal oxides capable of synergistic acid–base activation have shown outstanding performances on achieving glycerol conversions and GC selectivity upwards of 90% under mild, often solvent-free conditions [15,17]. Complementing experimental progress, density functional theory (DFT) has begun to shed light on the reaction mechanism, revealing that basic sites facilitate glycerol deprotonation, while Lewis acid centers polarize the carbonate, lowering energy barriers for nucleophilic substitution and cyclization to glycerol carbonate [17]. Nevertheless, mechanistic details specific to mixed metal oxide catalysis in alkyl carbonate routes remain underexplored [21].

This review thus aims to summarize recent developments in the glycerol to GC transformation via alkyl carbonates, focusing on the design and catalytic efficacy of mixed or promoter-modified metal oxide systems, with mechanistic understanding drawn from DFT studies. Through integrating performance data and theoretical insights, the goal is to define guiding principles for rational catalyst design and scalable process development.

### Transesterification of Glycerol Using Mixed/Promoted Metal Oxides and Mechanistic Insights from DFT

Among the various routes explored, the transesterification of glycerol with dimethyl carbonate (DMC) has emerged as one of the most promising methods for producing GC [22,23]. DMC is an environmentally benign, non-toxic, and biodegradable compound often referred to as a “green” reagent due to its low ecological impact and versatility as a methylating and carbonylating agent [24]. The benefit of using DMC is that it gives good atom economy, forming GC and methanol under mild operating conditions, with a simple downstream separation of volatile methanol. These attributes increase the overall sustainability and techno-economic attractiveness of the glycerol to GC production [23,25].

Catalyst selection plays a critical role in optimizing the reaction. Homogeneous base catalysts, such as alkali carbonates and hydroxides, have been widely studied, but issues of separation and waste generation have led to a shifted focus toward heterogeneous catalysts such as hydrotalcites and metal oxides (including CaO, ZnO, MgO, Mg-Fe, Mg-Al, and Mg/Al/Zr) which offer recyclability and improved sustainability [26,27]. Alvarez and coworkers [28] explored the continuous production of GC via the transesterification of glycerol with DMC using hydrotalcite-derived catalysts supported on suitable carriers [28]. The main goal was to overcome the drawbacks of batch processes, mass-transfer limitations, and catalyst recovery and to evaluate the potential of continuous fixed-bed systems for industrial application. The authors revealed that results were achieved under an optimized continuous-flow condition whereby the glycerol conversion and selectivity was found to be above 90% [28]. Overall, the transesterification of glycerol with DMC provides a technically and environmentally viable pathway for valorizing surplus glycerol, supporting both the economic sustainability of biodiesel production and the advancement of the bio-based chemical industry. The subsequent section will detail the studies based on the methods utilized for the preparation of mixed metal oxides in catalysis for improvement in the production of glycerol carbonate from glycerol.

## 2. Mixed Metal Oxides (MMOs)

### 2.1. Methods of Preparing Mixed Metal Oxides

**Figure 3:**
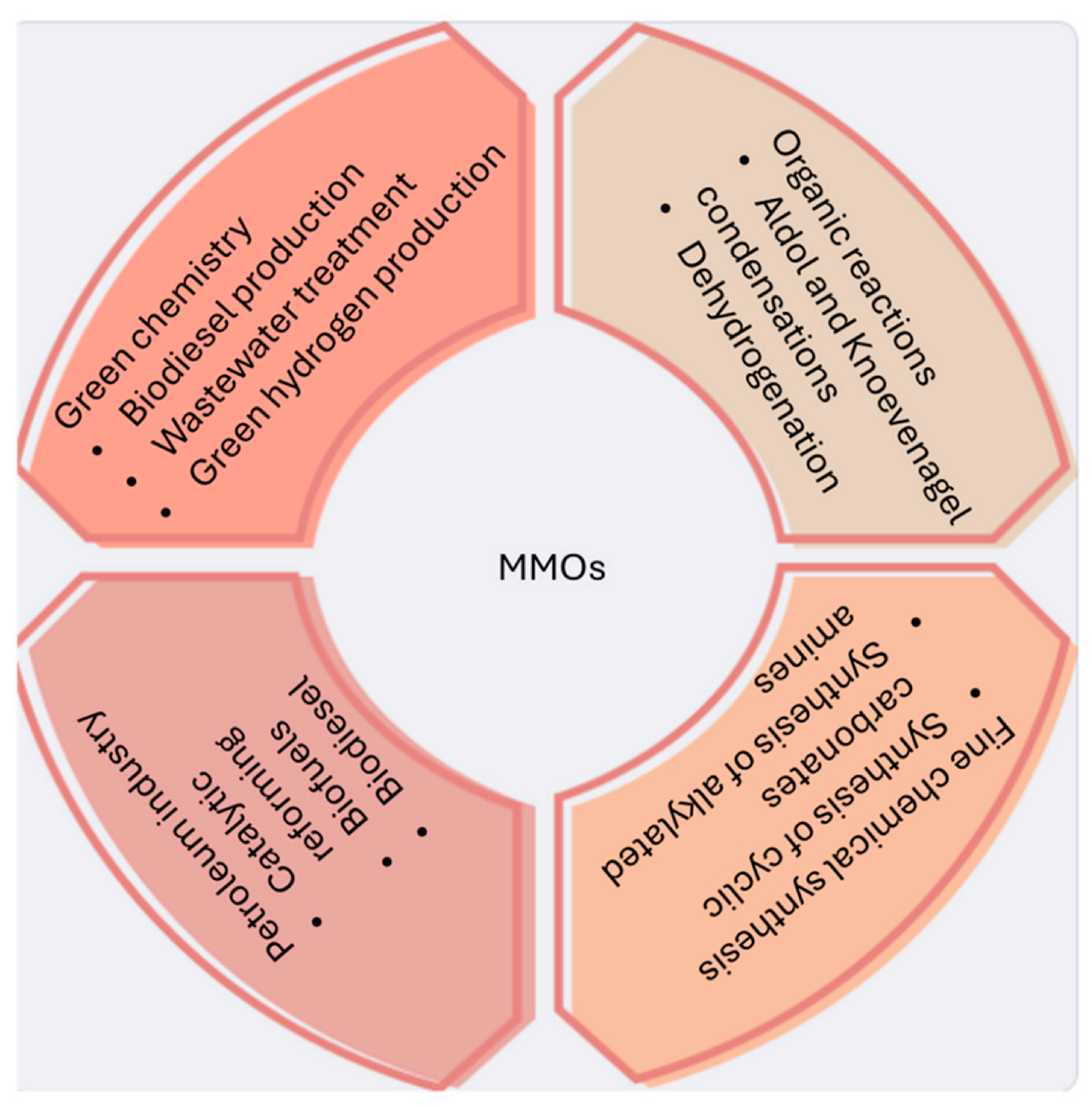
Mixed metal oxide applications.

Mixed metal oxides (MMOs) exhibit exceptional thermal stability, tunable acid–base properties, and strong metal–metal synergy, making them versatile in various applications [29]. They are widely employed as catalysts or supports in transesterification, oxidation, and reforming reactions, as well as in energy storage and environmental remediation. Their multifunctionality also extends to gas sensing and CO_2_ capture, positioning MMOs as key materials for sustainable and green chemical processes, as shown in Figure 3 [29]. MMOs can be synthesized via several established methodologies, with the choice of preparation route significantly influencing their crystallinity, surface area, acid–base site distribution, and ultimately their catalytic performance in glycerol conversion reactions. The following approaches are employed most often.

### 2.2. Hydrothermal Synthesis

Hydrothermal treatment enhances the crystallinity, particle uniformity, and textural properties of MMOs [30]. After coprecipitation, the slurry is transferred into a sealed autoclave and aged under hydrothermal conditions from 100 to 200 °C for several hours. This process improves structural ordering of the MMO and produces mixed oxides with controlled porosity upon calcination [30]. Hydrothermal methods are especially useful for synthesizing Mg-Zr, Mg-La, and ternary systems where precise metal incorporation is required [30]. Li et al. [31] developed and tested Mg-Zr mixed metal oxide catalysts prepared by a hydrothermal method for converting glycerol to glycerol carbonate via transesterification with DMC [31]. The study was based on comparing the hydrothermal method to a classical coprecipitation method, varying Mg-Zr ratios, optimizing reaction conditions, and evaluating catalyst stability and deactivation [31]. The authors claimed that the MMO materials prepared via hydrothermal preparation provided better dispersion, pore structure, and stability and were able to sustain activity even when recycled a few times [31].

### 2.3. Sol–Gel Method

The sol–gel process enables synthesis of highly homogeneous MMOs with tailored morphology. Metal alkoxides or salts are dissolved in aqueous/alcoholic solutions and subjected to hydrolysis–condensation reactions, forming a gel network containing the metal precursors. Subsequent drying (supercritical or conventional) and calcination generate finely dispersed oxide phases. Sol–gel-derived MMOs typically exhibit high surface area and uniform acid–base distributions, making them attractive for glycerol conversion. Lima-Corrêa et al. [32] prepared Mg-Al mixed oxides derived from a sol–gel hydrotalcite precursor and modified them in situ with small amounts of basic metals of K, Ba, Sr, La, or Li [32]. The study aimed to evaluate how those modifiers affect physicochemical properties and catalytic activity for ethyl transesterification [32]. Basic metal-modified Mg-Al mixed oxides showed significantly higher conversion and activity in ethyl transesterification tests than unmodified Mg-Al oxide [32,33]. The enhancement was correlated with the identity and loading of the added base metal. The sol–gel hydrotalcite route enables intimate dispersion of the dopant and host cations so that upon calcination, the resulting mixed oxides present tuneable basic properties. Even when textural parameters (surface area) were lowered, the creation of more and/or stronger basic sites drove higher transesterification rates [32,33].

### 2.4. Impregnation Technique

The impregnation method involves depositing secondary metal species onto a support or base oxide such as MgO, Al_2_O_3_, ZrO_2_, etc. [34]. Metal salts, especially nitrates, are dissolved in a suitable solvent and impregnated onto the support by wetness or incipient wetness methods [34]. After drying and calcination, well-dispersed metal oxide species are anchored on the support surface. The impregnation technique provides a versatile route to designing supported basic catalysts for glycerol transesterification [34,35]. By tailoring metal loading and support type, it is possible to optimize the balance between Brønsted and Lewis basic sites, ensuring high glycerol conversion and glycerol carbonate selectivity while maintaining catalyst reusability [35]. This approach is useful for tuning acid–base properties and incorporating redox-active metals, such as Fe, Cu, or Ce, into MMO systems. Dalma et al. [36] conducted a study that investigated the use of mixed oxides derived from calcined LDHs, typically Mg-Al, Mg-Zn, and Mg-Zr, as solid base catalysts for the transesterification of glycerol with DMC to produce GC [36]. In this study, introducing secondary metals, such as Zn, Zr, Cu, etc., via impregnation was utilized to modify the acid–base balance, improving both activity and stability [36].

The review conducted by Saeidabad and coworkers [37] provides a comprehensive overview of catalyst development for steam reforming of glycerol, a key route for hydrogen production from biodiesel-derived glycerol [37]. The authors emphasize how the choice of active metals, promoters, and supports governs catalyst performance, stability, and resistance to deactivation [36]. Importantly, alkali metals, such as K, Na, and Cs, enhanced the basicity of the material, particularly when prepared by the impregnation technique. Rare-earth promoters, such as Ce and La, also contribute to stability by improving oxygen storage and metal–support interactions.

### 2.5. Solution Combustion Synthesis (SCS)

**Figure 4:**
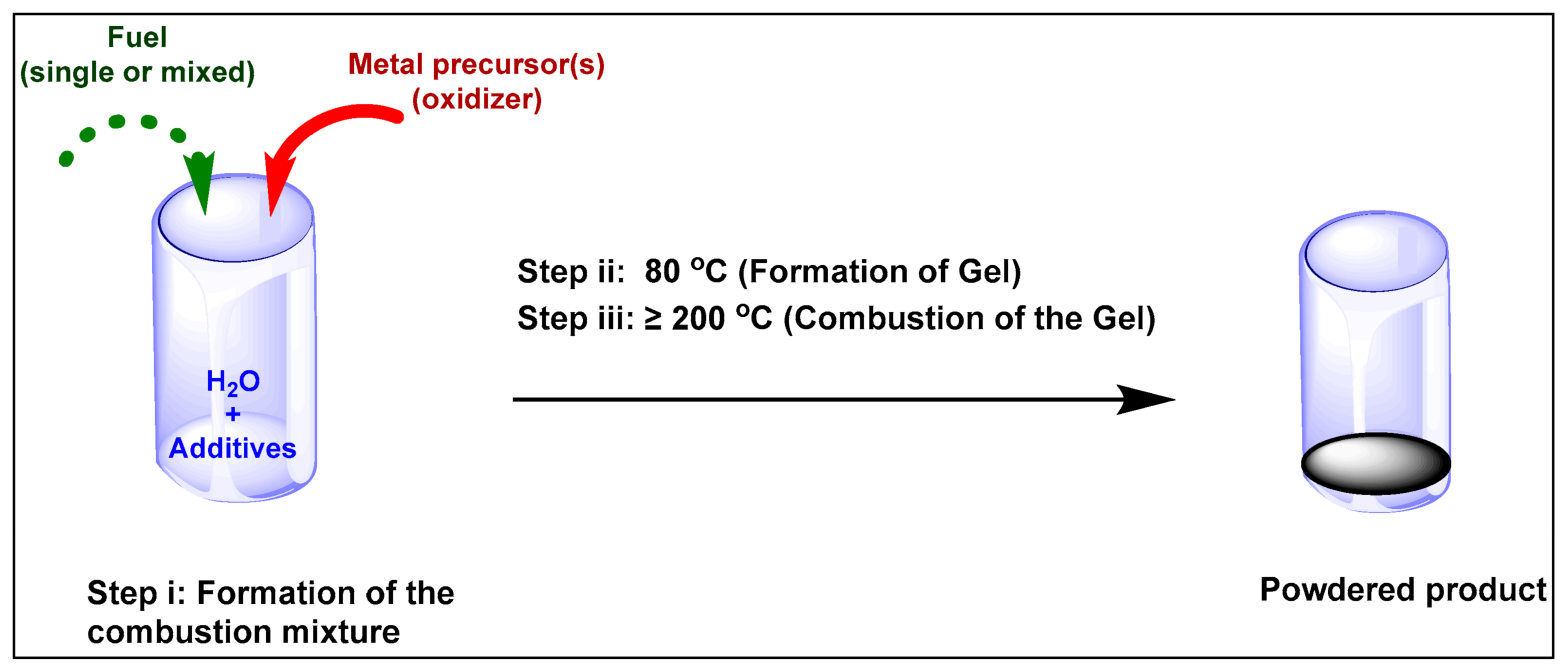
Schematic representation of the three fundamental stages involved in solution combustion synthesis [40].

The solution combustion method is a rapid, energy-efficient synthesis technique widely used for preparing mixed metal oxide catalysts and nanomaterials [38]. In this process, metal precursors, usually nitrates, are dissolved in an aqueous solution along with a fuel such as urea, glycine, citric acid, or hydrazine [38,39]. Upon heating, the mixture undergoes a self-sustained redox reaction between the oxidizing metal nitrates and reducing components. In the combustion synthesis route, metal nitrates act as oxidizers, and organic fuels, such as urea, glycine, and citric acid, serve as reductants. Upon ignition, a self-sustained exothermic reaction yields highly porous MMOs powders with nano-sized crystallites [39]. This technique produces MMOs with high surface area, tunable morphology, and strong metal–metal interactions. Padayachee et al. [40] described the process of solution combustion synthesis as typically proceeding through three main stages, which include precursor solution preparation, ignition and combustion, and product formation and cooling; see Figure 4. A study conducted by Hatab et al. [41] focused on designing Ni-based hybrid metal oxides via the solution combustion synthesis (SCS) route and evaluating their catalytic performance for the oxygen evolution reaction in alkaline electrolyte [41]. This study aimed to develop cost-effective alternatives to noble metals by exploiting the advantages of SCS in producing porous, nanostructured, and compositionally uniform oxides [41]. The authors emphasized that high dispersion of metal cations enhanced accessibility of catalytic sites [41].

### 2.6. Coprecipitation Method

The coprecipitation route is the most widely used technique for preparing MMO catalysts, particularly Mg-Al and Ca-Al systems derived from hydrotalcite precursors [42,43]. In this method, aqueous solutions of metal nitrates, chlorides, or sulfates are coprecipitated at constant pH (typically 9–11) using alkali solutions such as NaOH and Na_2_CO_3_ under vigorous stirring [43]. The precipitate is aged under controlled temperature (60–80 °C) to promote crystallization, followed by filtration, washing to remove residual ions, drying, and calcination (450–600 °C) [43]. Calcination decomposes the layered double hydroxide (LDH) precursor into homogeneous mixed oxides with high dispersion of metal cations and abundant basic sites [43,44]. Parameswaran et al. [10] performed a study pertaining to the transesterification of glycerol with dimethyl carbonate over Mg/Zr/Sr mixed oxide base catalysts [10]. A series of Mg/Zr/Sr mixed oxides prepared by coprecipitation with varying molar ratios was tested in glycerol and DMC via transesterification to form glycerol carbonate [10]. The study demonstrated that moderate-to-strong basic sites enhanced activity, whereas excessive basicity promoted the formation of glycidol as a side product. Overall, the work confirmed that optimized Mg/Zr/Sr mixed oxides are efficient and reusable heterogeneous catalysts for glycerol carbonate synthesis.

Rathod et al. [45] developed Mg-doped ceria-zirconia mixed oxide catalysts via a simple coprecipitation route and evaluated their efficiency in the Knoevenagel condensation of aromatic aldehydes with 2,4-thiazolidinedione. Structural analyses were fully characterized by XRD, FT-IR, SEM-EDS, CO_2_-TPD, and BET to confirm successful Mg incorporation, improved surface area, and enhanced basicity [45]. Among the prepared materials, the Ce_1_Mg_0.6_Zr_0.4_O_2_ catalyst exhibited the highest activity, delivering excellent yields of 90 to 96% under mild ethanol–water conditions. The study demonstrates that Mg doping effectively tunes surface basic sites and nanoscale properties, making these Ce-Zr-Mg oxides promising, reusable, and environmentally benign solid catalysts for efficient Knoevenagel condensations [45].

Furthermore, Dube et al. [25] demonstrated that MMOs prepared from hydrotalcite-like precursors show a close relationship between the hydroxyl groups located in the interlayer spaces of the LDH precursors and the surface oxygen species in the resulting oxides, which correspond to Brønsted and Lewis basic sites, respectively [25]. Moreover, surface hydroxyl groups enhance the accessibility of Brønsted-type basic sites by acting as charge-compensating anions, while calcination in an oxygen atmosphere promotes the generation of surface Lewis-type basic sites [25].

Among the various synthesis routes employed for the preparation of mixed metal oxides, each method has distinct advantages and limitations that critically influence the physicochemical properties of the resulting materials [30]. The hydrothermal method enables precise control over particle morphology, crystallinity, and size distribution under mild temperatures, yielding highly pure and well-crystallized products with tunable surface areas. However, it requires specialized autoclave equipment, involves lengthy reaction times, and offers limited scalability for industrial production [30]. Solution combustion synthesis is a rapid, energy-efficient, and cost-effective technique that produces nanocrystalline oxides with high surface areas in a single step without the need for elaborate equipment [39]. The exothermic nature of the process, however, makes it difficult to control particle size and homogeneity, and the use of fuel–oxidizer mixtures raises safety concerns [41]. The impregnation method is operationally simple and well suited for depositing active metal phases onto pre-formed supports with good reproducibility, but it offers poor control over metal dispersion [34,35]. This often results in an uneven distribution of active components and may require multiple impregnation–calcination cycles to achieve the desired loading [35]. The sol–gel method affords exceptional compositional homogeneity, high purity, and the ability to tailor porosity and surface properties at low processing temperatures [32]. This method makes it attractive for multi-component systems; its drawbacks include long gelation times and sensitivity to synthesis conditions. Other disadvantages include the use of expensive or toxic precursors, such as metal alkoxides, and significant volume shrinkage upon drying and calcination [32,33]. Finally, coprecipitation is a scalable, low-cost, and straightforward approach that ensures intimate mixing of metal cations at the atomic level, leading to homogeneous phase distributions and good catalytic activity [43,44]. Nevertheless, precise control of pH, temperature, and precipitation rate is essential to avoid phase segregation, broad particle size distributions, and the incorporation of residual counter-ions from the precipitating agents [44]. The selection of an appropriate synthesis method, therefore, depends on the target application, desired textural properties, available infrastructure, and the required degree of compositional and morphological control [44].

Having outlined all the methods that have been used for the preparation of MMOs, attention is now directed toward the preparation of MMOs through coprecipitation owing to their unique structural, textural, and chemical properties that allow them to function effectively in a variety of catalytic transformations [46]. The following section provides an overview of MMOs in glycerol conversion with DMC, indicating the synthesis strategies, physicochemical characteristics, and catalytic roles of these materials. In addition, the section will highlight how their composition and preparation methods influence performance. By establishing the fundamental principles and recent advances, this section aims to contextualize the subsequent discussion and underline the significance of MMOs in glycerol conversion with DMC in advancing sustainable catalytic processes. The subsequent section of this review will primarily examine studies on MMOs, quaternary LDH-derived MMOs, and waste-derived MMOs employed in the transesterification of glycerol.

#### 2.6.1. Mixed Metal Oxides in Glycerol Conversion with Dimethyl Carbonate

**Figure 5:**
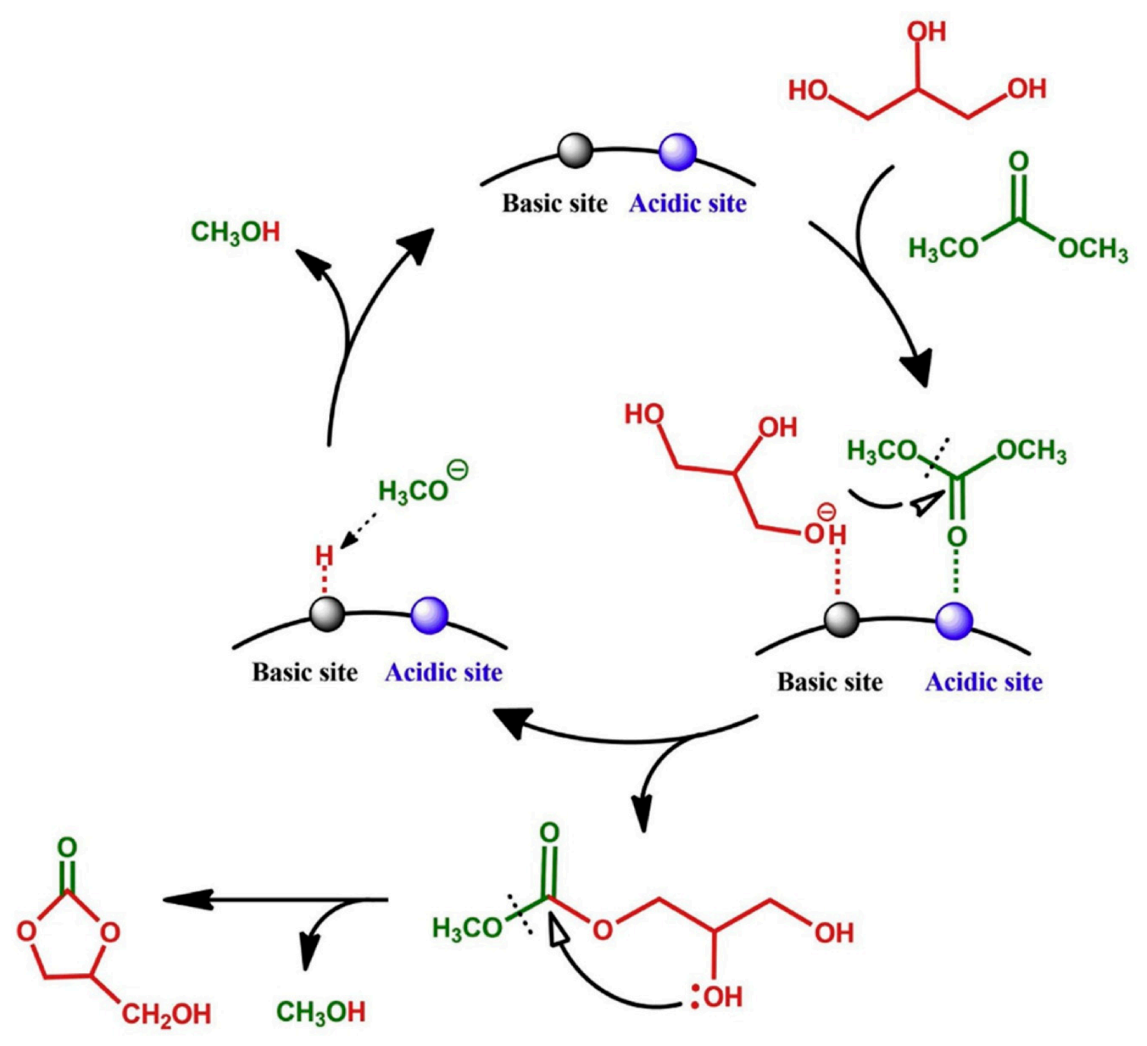
Mechanistic pathway of glycerol conversion to glycerol carbonate via transesterification. Reproduced with permission from ref. [47], Copyright (2020), Elsevier.

The conversion of GC with DMC to GC is strongly influenced by the choice of a catalyst. While homogeneous bases, such as NaOH and K_2_CO_3_, can achieve high activity, their use, as mentioned before, is limited by issues of separation, corrosivity, and waste generation. To address these limitations, MMOs have emerged as highly efficient heterogeneous catalysts due to their tunable acid–base properties, structural stability, and recyclability. Ultimately, in this process, the formation of glycerol GC is released from the catalyst surface, allowing the catalyst to be regenerated for subsequent cycles, as shown in Figure 5.

Liu et al. [48] prepared Mg-Al mixed oxide catalysts derived from hydrotalcite precursors, in which they demonstrated efficient GC formation under solvent-free or low-solvent conditions. Their catalysts showed good reusability with minor deactivation [48]. Zheng et al. [49] prepared CaAl hydrocalumite mixed metal oxide catalysts for the transesterification of glycerol [49]. The authors claimed that the best catalytic performance observed was for a Ca-rich catalyst with conversion of 82.4% and selectivity of 95.9% towards GC, thus showing a strong correlation between basicity and performance [49]. Pattanaik et al. [50] prepared Mg-Ba mixed oxide catalysts for continuous-flow systems in which a MgO:BaO molar ratio of 3:1 was achieved via coprecipitation. These catalysts exhibited up to 70% glycerol carbonate yield with nearly 100% selectivity [50]. The paper claims that the enhanced basicity was due to Mg^2+^ insertion into the BaO lattice, improving surface integrity and catalytic activity. Phadtare et al. [51] performed a study to investigate the use of a LaCoO_3_ perovskite-type oxide as a novel heterogeneous catalyst for the transesterification reaction [51], with the intention to explore if perovskites, as multifunctional oxides with both acidic and basic properties, can potentially surpass conventional mixed oxides [51]. In this study, LaCoO_3_ achieved GC conversions of above 90% with selectivity to GC of above 95% under optimized conditions. This was attributed to the catalyst exhibiting moderate basic sites from La^3+^-O^2−^ pairs and Lewis acidic sites from Co^3+^ which enabled simultaneous activation of GC and DMC [51].

Charif et al. [52] focused on a glycerol valorization study related to a green chemistry approach whereby the review concerns evaluated performance of various metal-oxide-based catalysts in the synthesis of GC [52]. Their main goal was to identify how different oxides contribute to the efficiency, selectivity, and sustainability of glycerol conversion [52]. Mg-Al oxides derived from hydrotalcites achieved high glycerol conversion above 90% and selectivity to GC above 95%. Incorporation of Zr, Ce, or Fe into Mg-based systems enhanced acid–base synergy and improved catalyst stability across multiple cycles [52].

Koranian et al. [53] explored the direct utilization of CO_2_ and glycerol to synthesize GC using metal oxide catalysts. This route is particularly attractive, since it integrates CO_2_ valorization with glycerol upgrading, aligning with the principles of green chemistry and carbon neutrality [53]. GC yields were generally moderate, 20–40%, under typical batch conditions. The authors also claimed that the incorporation of mixed oxides or bifunctional catalysts improved selectivity by simultaneously activating glycerol (via basic sites) and CO_2_ (via acidic sites) [53]. While MgO and CaO showed the highest activity due to their strong basicity, which promoted glycerol activation, ZrO_2_ and CeO_2_ contributed through Lewis acidic and redox-active sites, improving CO_2_ adsorption and activation. A balance of acid–base properties was essential for achieving higher GC selectivity [53].

In 2021, Praikaew et al. [53] investigated the production of GC from glycerol and DMC using CaO derived from waste eggshells as a sustainable heterogeneous catalyst [53]. A particular focus was given to process and energy intensification strategies for improving yield and efficiency, making the process more suitable for large-scale and green applications. This study showed that eggshell-derived CaO exhibited high basicity and good catalytic activity [53]. Glycerol conversion was above 90%, and GC selectivity was found to be 95% under optimized conditions. The study also confirmed that waste-derived CaO from eggshells is a promising, low-cost, and eco-friendly catalyst for GC production from glycerol and DMC. Moreover, process and energy intensification through microwaves, ultrasound, and continuous flow significantly enhanced productivity and reduced energy consumption [15,53].

Pradhan et al. [54] developed a sustainable and cost-effective process for the synthesis of GC from biowaste glycerol using pond ash, an industrial waste material, as a heterogeneous catalyst [54]. The work combined waste-to-value catalyst development with the valorization of glycerol, supporting a circular economy approach [54]. The findings of this study state that pond ash exhibited significant catalytic performance due to its basic and amphoteric sites. GC selectivity was 90%, and glycerol conversion of 95% was achieved under optimized conditions [54]. The process valorized two waste streams simultaneously, which are biowaste glycerol and industrial pond ash [54].

Another important avenue in glycerol valorization research involves the use of promoted metal oxides and enzymatic catalysts. Promoted metal oxides, obtained by incorporating alkali, alkaline earth, or transition metal promoters into conventional oxide frameworks, have been reported to enhance the basicity, redox behavior, and overall stability of the catalysts. These modifications often lead to higher glycerol conversions and improved selectivity toward value-added products such as GC by optimizing the balance between Brønsted and Lewis active sites. In contrast, enzymatic catalysts, especially lipases, represent a biologically inspired approach, offering high selectivity, mild operating conditions, and reduced environmental impact. Immobilization techniques have further improved their reusability and operational stability, making them a promising option for continuous biocatalytic processes. Together, these strategies highlight the complementary nature of inorganic catalyst promotion and biocatalysis, both aimed at overcoming the limitations of conventional systems and advancing greener, more efficient glycerol conversion technologies. The following section focuses on the gap in research on the subject and on the current literature based on promoted metal oxides and enzymatic catalyst systems as applied to the transesterification of glycerol to form GC.

#### 2.6.2. Promoted Metal Oxides and Enzymatic Catalysts in Glycerol Conversion

Beyond conventional MMOs, recent studies have focused on promoted metal oxide catalysts and enzymatic systems as alternative approaches to improve the efficiency and sustainability of glycerol conversion [55]. Promoted metal oxides are typically obtained by incorporating secondary metals or promoters, such as alkali, alkaline earth, or transition metals, into primary oxide frameworks to tune their acid–base properties, redox activity, and stability [55]. Such modifications have been shown to significantly enhance glycerol activation, selectivity toward glycerol carbonate, and resistance to deactivation. For instance, the addition of Zr, Ce, or alkali promoters into Mg^2+^- or Al^3+^-based oxides increases the synergy of active sites, allowing more efficient transesterification with DMC [11,56]. Table 1 shows some studies based on promoted metal oxides and enzymatic catalysts in glycerol conversion. Das and Mohanty (2019) [35] conducted a study and examined red mud (RM), an industrial waste, as a low-cost heterogeneous catalyst for converting renewable glycerol into GC using DMC [35]. The RM was promoted with K, Sr, and Mg to enhance its catalytic activity and stability [35]. The catalysts were synthesized by metal impregnation followed by calcination at around 800 °C. This study indicated that doping with K produced the strongest basic sites due to the formation of K_2_O species. Transesterification reactions were carried out using a DMC-to-glycerol molar ratio of approximately 2:1 under optimized conditions. Among all catalysts, the K-promoted sample calcined at 800 °C (RK-30–800) exhibited the best performance, achieving 94.8% glycerol conversion and 88.5% GC yield [35]. The authors found that the catalyst also showed stable reusability over four reaction cycles. Overall, the study demonstrated that K-modified red mud is an efficient, stable, and environmentally friendly catalyst for glycerol carbonate production [35].

Du et al. [57] also focused on producing GC from glycerol and DMC using a solid base catalyst composed of K_2_CO_3_ supported on magnesium oxide (K_2_CO_3_MgO) [57]. The catalyst was prepared by wet impregnation of K_2_CO_3_ onto MgO followed by drying and calcination. Under optimized conditions, which are a glycerol/DMC molar ratio of 2:1, 80 °C, 90 min reaction time, and 3 wt% catalyst loading, the result obtained was 96% glycerol conversion and 90% glycerol carbonate yield [57]. The catalyst maintained high activity and selectivity over several reuse cycles, indicating good stability. Overall, the K_2_CO_3_/MgO system proved to be an efficient, reusable, and environmentally friendly heterogeneous catalyst for sustainable GC production from renewable glycerol [57].

Dahdah et al. [58] investigated the effect of La promotion on Ni/Mg-Al hydrotalcite-derived catalysts used for the steam reforming of glycerol to produce hydrogen-rich gas [58]. The catalysts were synthesized via coprecipitation followed by calcination and reduction, with varying La loadings to evaluate structural and catalytic modifications [58]. Catalytic testing showed that La-promoted catalysts exhibited higher glycerol conversion and hydrogen yield compared to the unpromoted Ni/Mg-Al catalyst. The optimized La-Ni/Mg-Al sample achieved about 98% glycerol conversion and up to 80% H_2_ selectivity at 600 °C [58]. In addition, La addition improved coke resistance and catalyst stability over prolonged operation. The study concluded that lanthanum promotion strengthens the basicity and redox properties of Ni/Mg-Al hydrotalcite catalysts, resulting in enhanced performance and durability for hydrogen production from glycerol steam reforming [58].

Enzymes and enzyme-supported systems have been utilized as biocatalysts in the transesterification of glycerol with DMC for the production of glycerol carbonate. In parallel, enzymatic catalysts, particularly lipases, have attracted attention as green and selective biocatalysts for glycerol upgrading [59]. Enzymatic systems operate under mild reaction conditions, exhibit high selectivity, and minimize undesirable by-products [59,60]. Together, these two catalytic approaches highlight the growing diversity of strategies being explored to valorize glycerol. While promoted metal oxides emphasize activity, stability, and cost-effectiveness, enzymatic catalysts represent a biocompatible, eco-friendly alternative aligned with the principles of green chemistry. The following paragraphs detail the use of enzymes for the synthesis of GC from glycerol, the parameters used, and their corresponding results.

Gutierrez-Lazaro et al. [60] investigated the enzymatic synthesis of glycerol carbonate from glycerol and ethylene carbonate using lipase as a biocatalyst [60]. The research focused on understanding the effects of key operating variables, such as temperature, reaction time, enzyme concentration, and substrate molar ratio, on conversion efficiency and reaction kinetics [60]. The lipase-catalyzed reaction was conducted under mild conditions, and product formation was monitored using chromatographic analysis. Results showed that increasing temperature up to an optimal point of 60 °C enhanced glycerol conversion, while higher enzyme loading and longer reaction times further improved yield (1–100 h). Kinetic modelling revealed that the reaction followed a ping-pong bi-bi mechanism typical of lipase-catalyzed transesterification. Under optimized conditions, the process achieved approximately 95% glycerol conversion and around 90% GC yield [60]. The study concluded that enzymatic catalysis provides an efficient, selective, and environmentally benign alternative to conventional chemical routes for GC production [60].

Du et al. [61] developed an efficient biocatalyst for producing GC from glycerol and DMC using lipase immobilized on magnetic organosilica nanoflowers (MONFs) [61]. The nanoflower-like support was synthesized to provide a large surface area and good enzyme binding capacity. Lipase immobilization on MONFs improved enzyme stability, reusability, and catalytic efficiency compared to the free enzyme [61]. Reaction optimization showed that temperature, substrate ratio, and enzyme loading strongly influenced conversion [61]. Under optimal conditions at approximately 60 °C, a DMC/GL molar ratio of 2:1, and 6 h reaction time, the immobilized lipase achieved about 97% glycerol conversion and 93% GC yield. The catalyst retained over 85% of its activity after multiple reuse cycles. The study demonstrated that magnetic organosilica nanoflowers are promising supports for enzyme immobilization, offering a green, reusable, and high-performance system for sustainable GC synthesis [61].

Waghmare et al. [62] explored a study on ultrasound-assisted enzymatic process for synthesizing GC from glycerol and DMC using lipase as a biocatalyst [62]. The use of ultrasonic irradiation to enhance mass transfer, reduce reaction time, and improve product yield was investigated. Under optimized conditions at approximately 55 °C, a DMC/GLY ratio of 2:1, 3 wt% enzyme, and moderate ultrasound power, the process achieved about 98% glycerol conversion and 95% GC yield within a significantly shorter reaction time [62]. Ultrasound irradiation significantly shortened the reaction duration to approximately 4 h compared to 14 h required under conventional stirring conditions when catalyzed by Novozym 435. The lipase retained high activity over several reuse cycles, indicating minimal deactivation under ultrasonic conditions. In this study, the authors concluded that coupling enzymatic catalysis with ultrasound irradiation provides an efficient, energy-saving, and environmentally friendly method for producing GC from renewable glycerol [62]. The next sections discuss how the reaction parameters affect the transesterification process, highlighting their impact on glycerol conversion and GC selectivity.

**Table 1:** Summary of studies on promoted metal oxide and enzymatic catalysts for the conversion of glycerol to GC.

Catalyst Type/Composition	Promoter/ Enzyme Used	Preparation Method	Reaction Conditions	Key Findings	Glycerol Conversion (%)	GC Yield/ Selectivity (%)	Ref.
K-, Sr-, Mg-promoted red mud (RM)	K, Sr, Mg	Metal impregnation; calcination at 800 °C	DMC/glycerol = 2:1, 80 °C, 3 h	K-promoted RM exhibited highest basicity and activity	94.8	88.5	[35]
K_2_CO_3_/MgO	K_2_CO_3_	Wet impregnation, calcination	DMC/glycerol = 2:1, 80 °C, 90 min, 3 wt% cat.	Strong basic sites from K_2_CO_3_ improved glycerol activation	96	90	[57]
La-promoted Ni/Mg-Al hydrotalcite	La	Coprecipitation, calcination and reduction	Steam reforming of glycerol, 600 °C	La increased basicity and coke resistance; improved H_2_ yield and stability	98	80% H_2_ selectivity	[58]
Ce-, Zr-, Fe-promoted Mg-Al mixed oxides	Ce, Zr, Fe	Coprecipitation	Glycerol + DMC, 80 °C, 2 h	Promoters enhanced acid–base synergy and reusability	90–95	90–96	[52]
Lipase (free enzyme)	Lipase	Enzymatic catalysis	Glycerol + ethylene carbonate, 60 °C, 8 h	Mild, selective reaction; optimized at 60 °C with high yield; followed ping-pong bi-bi kinetics	95	90	[60]
Lipase immobilized on magnetic organosilica nanoflowers (MONFs)	Lipase	Enzyme immobilization	DMC/glycerol = 2:1, 60 °C, 6 h	Immobilized lipase enhanced stability	97	93	[61]
Ultrasound-assisted lipase catalysis	Lipase	Enzymatic + ultrasound	55 °C; 2:1 DMC/glycerol; 3 wt% enzyme	Ultrasound improved mass transfer; reduced time	98	95	[62]

## 3. Factors Influencing Performance

High glycerol conversion and selectivity can be achieved by carefully optimizing the key factors influencing the reaction. Critical parameters, such as catalyst dosage, glycerol to DMC molar ratio, reaction temperature, reaction time, and solvents, significantly affect the adsorption, desorption, and surface reaction rates during the transesterification of glycerol with DMC. Researchers have utilized various statistical approaches to optimize these conditions, as indicated in Table 2. In most studies, a one-variable-at-a-time method was used in which the effect of each individual parameter on glycerol conversion and glycerol carbonate selectivity was evaluated while keeping all other variables constant.

### 3.1. Reaction Temperature

Based on kinetic theory, reaction temperature plays a crucial role in determining the rate of any chemical reaction. As an independent variable in the reaction pathway, it is essential to maintain an optimal temperature to achieve maximum efficiency. The relationship between temperature and reaction rate is described by the Arrhenius equation, which expresses how reaction rates increase with rising temperature [69]. The transesterification of glycerol to produce glycerol carbonate is a reversible process in which the forward reaction leads to the formation of glycerol carbonate, and this reaction is known to be a non-spontaneous and endothermic reaction [69,70,71]. Therefore, a higher reaction temperature is necessary to drive the process in the forward direction. In such non-spontaneous reactions, additional thermal energy is required to overcome the resistance caused by the viscosity of the reactant mixture, especially in heterogeneous catalytic systems [71]. Therefore, a higher reaction temperature is essential to improve the miscibility between the DMC and glycerol phases in the presence of a solid catalyst, thereby facilitating the surface reactions that lead to glycerol carbonate formation. In essence, determining the optimal reaction temperature largely depends on the type of carbonate source used [9,72]. Additionally, factors such as the thermal sensitivity and physicochemical properties of the carbonate source, particularly its boiling point, are crucial considerations in selecting the appropriate reaction temperature. The transesterification of glycerol with dimethyl carbonate (DMC) or diethyl carbonate typically requires a reaction temperature in the range of 65–150 °C to obtain high yields of GC [73].

A study conducted by Koranian et al. [74] was based on MMO catalysts containing Mg, Ca, and Al for converting glycerol into GC [74]. The catalysts were synthesized via coprecipitation. The influence of reaction temperature on glycerol conversion and GC yield was a key focus. Results showed that increasing the temperature enhanced the miscibility of reactants and promoted the transesterification reaction [74]. Optimal temperatures led to the highest glycerol conversion and GC selectivity, with the best-performing catalyst achieving approximately 95% glycerol conversion and 91% glycerol carbonate yield. The study concluded that the temperature strongly affects catalytic performance, and careful optimization is essential to maximize GC production using Mg-Ca-Al mixed oxide catalysts [74].

Pradhan et al. [75] report the study of the promotional effects of transition metals (Cr and V) on MgO catalysts for synthesizing GC from glycerol and DMC [75]. The catalysts were prepared by impregnating MgO with varying amounts of Cr or V. Results indicated that increasing the reaction temperature from 70 to 150 °C enhanced reactant miscibility and the rate of transesterification, leading to higher glycerol conversion and selectivity toward GC. Among the tested catalysts, Cr-promoted MgO exhibited the best performance, achieving approximately 98% glycerol conversion and 90% GC yield at the optimal temperature [75].

### 3.2. Catalyst Dosage

A catalyst is essential in kinetically inert reactions, as it provides an alternative reaction pathway with a lower activation energy. In heterogeneous catalysis, an effective catalyst must offer numerous active sites that can adsorb reactant molecules, positioning them to promote bond-breaking and bond-forming processes, ultimately facilitating product formation [11]. Increasing the catalyst dosage proportionally raises the number of active or basic sites, thereby improving the efficiency of the transesterification reaction. The reaction of glycerol with DMC requires a catalyst with basic sites, which help abstract a proton from a glycerol hydroxyl group and initiate the formation of GC [1,69]. While both homogeneous and heterogeneous catalysts can facilitate this transesterification, heterogeneous catalysts are generally favored due to their lower downstream processing costs.

The effect of catalyst loading has also been examined relative to the mass of glycerol used [12]. At elevated catalyst loadings, GC may decompose via decarboxylation to produce glycidol, which decreases the overall GC yield [76]. Parameswaram et al. [77] detailed a study on magnesia–ceria (MgO-CeO_2_) mixed oxide catalysts for the selective transesterification of glycerol with DMC to produce GC [77]. The catalysts were prepared via a coprecipitation method. The effect of catalyst composition and loading on glycerol conversion and GC yield was a key focus. Results showed that increasing the MgO content enhanced the number of basic sites, promoting higher glycerol conversion and selectivity toward GC. The optimal MgO-CeO_2_ catalyst achieved approximately 96% glycerol conversion and 92% GC yield [77]. The authors concluded that the catalyst’s composition and loading critically influence product formation, with well-balanced magnesia–ceria catalysts providing high activity and selectivity for glycerol carbonate synthesis. Increasing the catalyst loading generally enhances the number of available active or basic sites, which improves glycerol adsorption and promotes the transesterification reaction [43]. However, beyond an optimal loading, excessive catalyst can lead to side reactions, such as decarboxylation of glycerol carbonate to glycidol, ultimately reducing the GC yield [78].

### 3.3. Reaction Time

Reaction time is a crucial parameter that significantly influences the transesterification of glycerol with DMC. The kinetically active reaction proceeds rapidly, requiring a shorter time to reach completion [79]. In a heterogeneous system, reaction time plays a vital role in determining the efficiency and yield of GC formation during the transesterification of glycerol with DMC. It governs the extent of interaction between the reactants and influences how quickly the system reaches equilibrium under given conditions [80]. Short reaction times may lead to incomplete conversion of glycerol, resulting in lower product yield, while excessively long reaction times can promote undesirable side reactions that reduce selectivity toward glycerol carbonate [80,81]. Therefore, optimizing reaction time is essential to balance conversion efficiency and product purity. In heterogeneous catalytic systems, reaction time is further affected by the diffusion and miscibility of reactants at the catalyst interface, which can limit the overall reaction rate [82]. The kinetic nature of the transesterification process determines how rapidly equilibrium is achieved, emphasizing the need for precise control of reaction duration [83]. Thus, reaction time remains a critical parameter for achieving high catalytic performance and energy-efficient production of glycerol carbonate.

Praikaew et al. [84] conducted a study on process and energy intensification of glycerol carbonate production from glycerol and dimethyl carbonate in the presence of eggshell-derived CaO heterogeneous catalyst [84]. The investigation was based on the transesterification of glycerol with DMC using a calcium oxide (CaO) catalyst derived from waste eggshells [84]. The methodology involved calcining eggshells at high temperatures to obtain active CaO, which was then used in a batch reactor system under varying reaction conditions [84]. Initially, the yield increased with longer reaction time due to improved contact between reactants and catalyst surface, but after an optimal duration, further extension led to a decrease in yield, likely caused by side reactions or product degradation [84]. The study identified an optimum reaction time of around 90 min, with a maximum GC yield of approximately 97% under mild conditions at 80 °C, 10 wt% catalyst loading, and 1:2 glycerol-to-DMC ratio [84].

### 3.4. Glycerol: DMC Molar Ratio

Glycerol: DMC molar ratio and solvent effects have been studied and explored through the transesterification of glycerol. In the literature, most studies have stated that the transesterification process follows a stoichiometric ratio of one mole of carbonate reacting with one mole of glycerol to yield one mole of GC and one or two moles of by-products, which may include methanol, ethanol, ethylene glycol, or propylene glycol, depending on the carbonate used [1,24]. However, because glycerol is hydrophilic and most carbonate sources are hydrophobic, the reactants are poorly miscible, and the reaction remains reversible [69]. To overcome these limitations and drive the reaction toward greater GC formation, an excess of carbonate is typically introduced to improve both conversion efficiency and product yield [85]. Two-phase separation between the reactants can be avoided either by using a very large amount of carbonate, which functions both as a reactant and as a solvent, or by introducing an appropriate organic solvent to enhance miscibility [85,86].

In certain cases, an organic solvent is introduced to compensate for a low carbonate-to-glycerol molar ratio. The reaction between the highly polarized glycerol and aprotic carbonates, such as DMC or DEC, can be significantly improved by using polar aprotic solvents, like dimethylformamide (DMF), dimethyl sulfoxide (DMSO), or acetonitrile, or hydrophilic solvents, such as tetrahydrofuran (THF), tert-butanol, ethanol, or methanol, which enhance the solubility of the reactants [69]. For example, the addition of suitable polar solvents, such as DMF or DMSO, dramatically increased glycerol carbonate (GC) yield from 17% to 99% in transesterification catalyzed by uncalcined Mg/Al hydrotalcite [65]. Glycerol conversion was found to correlate with solvent polarity, decreasing in the order of DMSO (50%) > DMF (30%) > DMA (20%) [65]. However, inappropriate solvent selection can result in unwanted by-products, including glycidol and other unidentified compounds, as also observed with ethanol. These findings underscore the critical role of solvent polarity in influencing both glycerol conversion and product selectivity in the transesterification process [87,88].

Álvarez et al. [28] conducted a study on the synthesis of glycerol carbonates by transesterification of glycerol in a continuous system using supported hydrotalcites as catalysts. They investigated the continuous production of GC from glycerol and DMC using supported hydrotalcite-based catalysts in a fixed-bed flow reactor. The experimental approach evaluated the influence of various solvents, operating temperatures, and flow rates on the catalytic activity, selectivity, and stability of the system [28]. The results demonstrated that the solvent type had a significant impact on glycerol conversion and GC yield. When polar aprotic solvents such as acetone and acetonitrile were used, they enhanced the miscibility between glycerol and DMC, improving mass transfer and catalyst–reactant interaction [28]. This resulted in glycerol conversions exceeding 90% and glycerol carbonate yields above 85% under optimized continuous-flow conditions. In contrast, nonpolar solvents or solvent-free systems exhibited lower conversions, below 60%, due to phase separation and poor diffusion across the catalyst surface [28]. Additionally, the use of appropriate solvents contributed to greater catalyst stability and reduced deactivation during extended operation [28]. Overall, the study concluded that solvent selection is a crucial factor in enhancing process efficiency, as it directly affects reaction kinetics, product yield, and catalyst longevity.

Recently, density functional theory (DFT) studies have provided some valuable insights into the transesterification of glycerol with carbonates, confirming a consensus reaction mechanism for glycerol carbonate formation. These computational investigations clarify the reaction pathways, intermediate species, and energy profiles, supporting experimental observations and helping to rationalize factors that influence catalyst activity, selectivity, and product yield. By combining theoretical and experimental approaches, DFT studies have strengthened the understanding of the molecular-level processes governing glycerol carbonate synthesis. The following section reviews studies on the use of DFT in the transesterification of glycerol.

## 4. Density Functional Theory (DFT) and Mechanistic Studies

Historically, there is little information on mechanistic insight and DFT utilization for the transesterification of glycerol using DMC. Recently, DFT studies on glycerol transesterification to GC aimed to understand reaction mechanisms, optimize catalysts, and improve reaction conditions by modelling the interactions between glycerol, the carbonate source (such as CO_2_, DMC, urea, and various alkylene carbonates like ethylene carbonate and propylene carbonate), and the catalyst at a molecular level. These studies help explain how factors like catalyst basicity, reaction temperature, and reactant ratios influence conversion and yield.

For example, DFT has been used to investigate the role of basic sites in K-modified silicalite-1 catalysts and the catalytic mechanisms on oil palm ash [16]. Nyepetsi et al. [89] conducted a study on the carbonate-catalyzed transesterification of sunflower oil for biodiesel production using in situ monitoring and DFT calculations. The investigation was based on the mechanism and kinetics of biodiesel synthesis from sunflower oil using carbonate-based catalysts. The methodology combined experimental in situ spectroscopic monitoring with DFT computational analysis to understand the reaction pathway and identify active intermediates [89]. Real-time Fourier-transform infrared (FTIR) spectroscopy was employed to track the progress of the transesterification reaction and quantify product formation under controlled conditions. The results demonstrated that carbonate catalysts effectively promoted the conversion of triglycerides to fatty acid methyl esters (biodiesel) with high efficiency and selectivity [89]. DFT calculations revealed the stepwise mechanism of the reaction, highlighting the role of carbonate ions in activating methanol and facilitating the nucleophilic attack on the carbonyl carbon of triglycerides [89].

Adjieufack et al. [90] performed a study on the reaction mechanism of glycerol carbonate decomposition by bond evolution theory. Authors employed DFT combined with bond evolution theory (BET) to analyze the electronic and structural changes occurring during the decomposition of GC [90]. This computational approach allowed for a detailed examination of bond-breaking and bond-forming events throughout the reaction pathway [90]. The topological analysis identified distinct transition states and intermediates, clarifying the stepwise mechanism of GC decomposition [90]. The results revealed that the process involves the cleavage of the C-O and C-C bonds, leading primarily to the formation of glycidol and carbon dioxide (CO_2_) as the main products. Overall, the study demonstrated that BET provides a powerful framework for understanding the electronic rearrangements and energetic profiles underlying the thermal decomposition of glycerol carbonate, offering insights that complement experimental findings and aid in the design of more stable reaction systems [90].

Luo et al. [91] conducted a study on unveiling the complementary mechanism in the one-pot synthesis of GC from CO_2_ and glycerol, combining experimental catalysis and DFT calculations to explore the detailed mechanism of GC formation directly from glycerol and CO_2_ [91]. The methodology involved conducting one-pot catalytic reactions under varying conditions and using computational modelling to identify the reaction intermediates, energy barriers, and pathways [91]. The results revealed that the synthesis proceeds through a complementary dual-path mechanism where both carbonate and glycidol intermediates are involved [91]. The study confirmed that the synergy between experimental and theoretical findings provides a clear understanding of the reaction route and supports the development of efficient catalytic systems for direct CO_2_ utilization in glycerol carbonate production.

Nan et al. [92] also performed a study combining experimental and quantum chemical calculations on the high-efficiency transesterification of DMC with alcohol catalyzed by CaO. They investigated the catalytic performance and reaction mechanism of CaO-catalyzed transesterification between DMC and various alcohols [92]. The methodology integrated experimental kinetic studies with quantum chemical (DFT) calculations to analyze the reaction pathway, activation energies, and intermediate structures [92]. The results demonstrated that CaO exhibits excellent catalytic activity and stability, achieving high conversion rates and selectivity toward the desired alkyl carbonates under mild reaction conditions [92]. Computational findings revealed that the active surface oxygen species on CaO facilitate methoxy group abstraction and nucleophilic attack by the alcohol, lowering the overall energy barrier and enhancing reaction efficiency [92]. Overall, the combination of experimental data and theoretical modelling confirmed the superior catalytic performance of CaO and provided molecular-level insight into its mechanism in DMC transesterification.

## 5. Research Gaps and Future Scope

Despite significant progress in the catalytic conversion of glycerol to GC, several critical research gaps remain.

- Mechanistic understanding through DFT is still limited, particularly for complex mixed and promoter-modified metal oxides, where the precise nature of active sites and reaction intermediates remains unclear.
- The structure–activity relationship governing how synthesis methods, metal ratios, and promoter additions influence acid–base balance and catalyst durability is not yet fully established.
- Additionally, a lack of standardized reaction conditions and kinetic modelling hinders meaningful comparison among studies, while the role of solvent polarity and carbonate excess in controlling miscibility and selectivity requires deeper mechanistic exploration.

Although progress has been made with waste-derived and promoted catalysts, more work is needed to design cost-effective, scalable, and sustainable systems with minimal energy demand. Enzymatic and hybrid catalytic systems, though promising for their selectivity and mild conditions, remain underdeveloped and require further optimization for industrial application.

Looking ahead, future research should integrate experimental and theoretical approaches to build a unified understanding of the transesterification mechanism at the molecular level. The application of advanced in situ characterization techniques, such as DRIFTS, XPS, and Raman spectroscopy, alongside DFT-based modelling, can provide real-time insights into active site evolution and reaction pathways. Moreover, the development of bifunctional and waste-derived catalysts, combined with process intensification strategies, such as microwave, ultrasound, or continuous-flow systems, could enhance reaction efficiency and energy utilization. Finally, establishing predictive computational frameworks to guide catalyst design and exploring hybrid inorganic–biocatalytic systems represent promising directions toward sustainable, high-yield glycerol carbonate production aligned with green chemistry principles.

## 6. Conclusions and Future Perspectives

The conversion of glycerol to GC via transesterification with alkyl carbonates has emerged as a sustainable and economically viable pathway for valorizing the biodiesel by-product glycerol. Evidence from the literature confirms that mixed and promoter-modified metal oxides, such as Mg-Al, Mg-Zr, Ca-Ce, Mg-Fe, and Mg-Ba systems, exhibit exceptional activity and selectivity, often achieving over 90% glycerol conversion and 95% GC selectivity under mild or solvent-free conditions. Their superior performance arises from the synergistic balance of Lewis acidic and Brønsted basic sites, which facilitate both glycerol deprotonation and carbonate activation. Furthermore, process intensification techniques, such as microwave heating, ultrasound, and continuous flow, have enhanced efficiency, reduced energy consumption, and improved scalability. Complementary DFT studies have provided molecular-level insight into the reaction mechanism, confirming base-assisted deprotonation and carbonate polarization as key steps in GC formation. However, the integration between computational modelling and experimental validation remains limited, highlighting the need for more unified approaches.

Despite these advances, several research gaps persist. There is insufficient understanding of how catalyst preparation methods, metal interactions, and promoter incorporation influence acid–base properties, reaction kinetics, and catalyst longevity. Similarly, the influence of solvent polarity, reactant miscibility, and operating parameters requires more systematic investigation. The potential of waste-derived materials and enzymatic or hybrid catalytic systems also remains underexplored despite their promise for green and circular chemical processes.

Looking forward, future studies should aim to bridge experimental and theoretical insights to establish a comprehensive mechanistic understanding of glycerol transesterification. The exploration of integrating biocatalysis with heterogeneous catalysis could also open new avenues for low-energy, selective, and sustainable GC production, supporting the broader transition toward a circular and carbon-neutral bioeconomy.

## Figures and Tables

**Table 2:** Summary of studies on factors influencing performance in the transesterification of glycerol with dimethyl carbonate using heterogeneous catalysts.

Factor Studied	Catalyst Type/Composition	Reaction Conditions	Glycerol Conversion (%)	GC Selectivity/Yield (%)	Key Finding/Remark	Ref.
Reaction temperature	Mg/MnO_2_ and Mg/CuO heterogeneous catalyst	50–100 °C	97	94	Temperature enhances miscibility and reaction kinetics	[22]
Catalyst loading	Ca-Al hydrocalumite	1–15 wt% catalyst	97	93	Optimal loading essential for balancing activity and selectivity	[49]
Reaction time	Ceria-based catalyst	1–7 h	97	97	Optimum time (6 h) ensures high yield	[63]
Glycerol:DMC molar ratio	CaO	1:1–1:7	80–95	85–96	Excess carbonate compensates for immiscibility and reversibility	[64]
Solvent effect	Supported hydrotalcite	Polar solvents	>90	>85	Solvent polarity is crucial for efficient transesterification	[65]
Solvent polarity	Ca/Al mixed oxides derived from hydrocalumite	DMF and DMSO	90 DMF and DMSO	91 DMF and DMSO	Highly polar solvents boost glycerol solubility and GC yield	[66]
Catalyst basicity/acidity	Fe-La mixed oxide catalysts	Coprecipitation	71	>85	Acid–base synergy is key for high turnover and catalyst stability	[67]
Promoter effect	Potassium-modified silicalite-1	Impregnation	96	95	Promoter incorporation enhances catalytic strength and lifetime	[68]

## Data Availability

No new data were created or analyzed in this study. Data sharing is not applicable to this article.
